# A case of benign multicystic peritoneal mesothelioma

**DOI:** 10.1093/jscr/rjae629

**Published:** 2024-10-10

**Authors:** Jessica Wassef, Jane Kim, Tasneem Farag, Hassan Masoudpour, George Tsioulias

**Affiliations:** Department of Surgery, Englewood Hospital and Medical Center, Englewood, NJ 07631, United States; Department of Surgery, Englewood Hospital and Medical Center, Englewood, NJ 07631, United States; Department of Surgery, Hackensack Meridian Health Palisades Medical Center, North Bergen, NJ 07047, United States; Ain Shams University, Faculty of Medicine, Cairo 4240310, Egypt; Department of Surgery, Englewood Hospital and Medical Center, Englewood, NJ 07631, United States; Department of Surgery, Englewood Hospital and Medical Center, Englewood, NJ 07631, United States

**Keywords:** mesothelioma, peritoneal cyst, cytoreductive surgery, hyperthermic intraperitoneal chemotherapy

## Abstract

Benign Multicystic Peritoneal Mesothelioma (BMPM) is an exceedingly rare benign abdominal neoplasm with fewer than 200 cases reported worldwide. Owing to its rarity, vague clinical picture, and elusive causes, diagnosis is often delayed or missed. Histopathological examination and immunohistochemical staining are crucial for definitive diagnosis. However, due to lack of substantial literature, the standard of care and future prognosis remain subjects of inquiry. We present a case report of one female patient diagnosed with BMPM and treated with surgical resection.

## Introduction

Benign Multicystic Peritoneal Mesothelioma (BMPM) is a rare neoplasm originating from the peritoneum, primarily affecting premenopausal females and rarely men. The objective is to delineate the diagnostic approach and therapeutic strategies for BMPM.

## Case report

**Figure 1:**
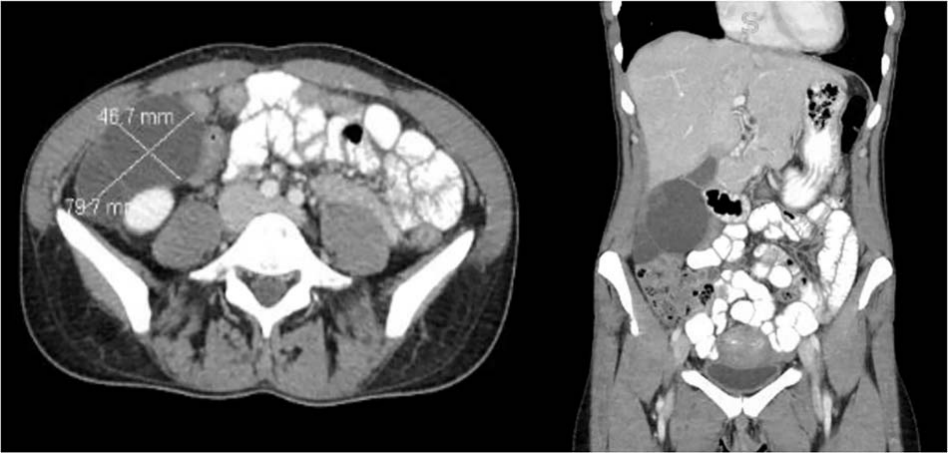
CT showing cystic structure abutting the right kidney, liver, and gallbladder.

**Figure 2:**
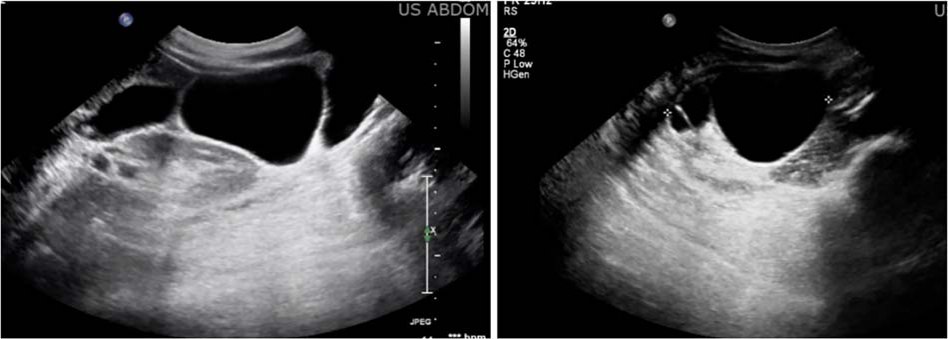
Ultrasound imaging revealing a multiseptated cystic mass measuring 4.4 × 7.7 × 9.3 cm, suspicious for exophytic complex hepatic cyst.

A 47-year-old female with no significant past medical history presented with right lower quadrant pain radiating to the back, which had persisted for 3 days. Additionally, she reported a 6 pound weight loss over the course of one year. She had no personal or family history of malignancy. Her labs were within normal limits and her CEA, CA 19–9, CA 125, and CA 27.29 were not elevated. A CT scan of the abdomen and pelvis (Fig. 1) revealed a multilobulated, multiseptated, well circumscribed cystic mass measuring 8.0 × 4.7 × 7.9 cm in size abutting the inferior aspect of the gallbladder extending anteriorly to the right kidney into the paracolic gutter. Ultrasound imaging (Fig. 2) revealed as multiseptated cystic mass measuring 4.4 × 7.7 × 9.3 cm, suspicious for exophytic complex hepatic cyst. No mural nodularity or abnormal mural hypervascularity was seen. Tumor markers LDH, CA 125, and CEA were within normal limits. The decision was made to perform a diagnostic laparoscopy with potential resection.

Intraoperatively, the cyst was noted to be extraperitoneal with adhesions to the peritoneum of the gallbladder, hepatic flexure of the colon, right colon, and small bowel. Adhesions were meticulously dissected and complete resection of the lesions was performed. The patient experienced an uneventful postoperative recovery.

Histopathological analysis confirmed benign multicystic mesothelioma demonstrating expression of pankeratin CAM 5.2 as well as mesothelial cell markers, including calretinin and WT-1. However, it tested negative for pax 8, ER, CD34, and CD31.

## Discussion

Unlike malignant mesothelioma, the etiology of BMPM remains elusive. Three hypotheses have been suggested. The first proposed hypotheses include association with chronic inflammation stemming from various sources such as previous surgeries, endometriosis, pelvic inflammatory disease, peritoneal dialysis, or Familial Mediterranean fever (FMF); although in cases of FMF, malignant mesothelioma is generally more common [1]. The second hypothesis suggests a non-inflammatory, purely neoplastic transformation of peritoneal mesothelium [2]. Lastly, a hormonal hypothesis has been suggested corroborated by the high sensitivity of the tumor to hormones. Some BMPM may be positive for estrogen and progesterone receptors. This is observed in the higher incidence in females of reproductive age as well as response to selective estrogen receptor modulators (SERM) such as tamoxifen and gonadotropin-releasing hormone analogs [3, 4].

Typically presenting with nonspecific symptoms, BMPM diagnosis is often delayed. Clinical manifestations arise from mass effect and include abdominal or pelvic pain, distension, altered bowel habits, unintentional weight gain, or intestinal obstruction. On examination, there may be abdominal tenderness, distension or palpable masses. A rare case was reported wherein the patient presented with a picture of appendicitis [5].

Laparoscopy is the best way to obtain definitive diagnosis and to evaluate the extent of disease. High Ca 19–9 serum concentrations are found to be associated with BMPM [6]. MRI is preferred for imaging due to its superior differentiation capability, yet laparoscopy allows for direct visualization and histopathological confirmation [3]. BMPM will often test positive for WT-1 and calretinin.

Important differentials include malignant peritoneal mesothelioma (MPM), cystic adenomatoid tumor, and lymphangioma. Malignant peritoneal mesothelioma, notability associated with asbestos exposure, typically presents with abdominal pain and weight loss. Lymphangiomas exhibit characteristic features such as chylous fluid and positive CD2–40 staining. It does not stain positive for mesothelial markers [7, 8].

Complete resection of the tumor is the most common treatment, although due to a significant recurrence rate and potential for malignant transformation, alternative approaches involving cytoreductive surgery (CRS) with hyperthermic intraperitoneal chemotherapy (HIPEC) have been proposed, offering favorable long-term outcomes. Studies have suggested it can prolong survival with 80% remaining disease free after 10 years [3, 9, 10]. Reduction in recurrence rates can be owed to removal of microscopic residual neoplastic cells. In an attempt to detect relapses early, some authors suggest implementing post-operative follow up CT scans every 3 months for the first year then annually for the following five years [11]. However, whether this improves outcomes is yet to be established. Because of the rarity of BMPM, prognosis is not certain although it appears to be favorable with only one reported fatality [12].

## Conclusion

BMPM is a rare neoplasm which poses diagnostic challenges due to its resemblances to other cystic lesions. Although no consensus exists regarding optimal diagnostic or therapeutic strategies, complete resection remains the preferred approach, with CRS-HIPEC as a viable option to mitigate recurrence in patients with borderline histopathology.
